# Assessment of algorithms for high throughput detection of genomic copy number variation in oligonucleotide microarray data

**DOI:** 10.1186/1471-2105-8-368

**Published:** 2007-10-02

**Authors:** Ágnes Baross, Allen D Delaney, H Irene Li, Tarun Nayar, Stephane Flibotte, Hong Qian, Susanna Y Chan, Jennifer Asano, Adrian Ally, Manqiu Cao, Patricia Birch, Mabel Brown-John, Nicole Fernandes, Anne Go, Giulia Kennedy, Sylvie Langlois, Patrice Eydoux, JM Friedman, Marco A Marra

**Affiliations:** 1 Genome Sciences Centre, BC Cancer Agency, British Columbia Cancer Agency, Suite 100, 570 West 7th Avenue, Vancouver, BC, V5Z 4S6, Canada; 2 Affymetrix Inc., 3420 Central Expressway, Santa Clara, CA 95051, USA; 3 Dept. of Medical Genetics, University of British Columbia, Children's & Women's Hospital, Box 153, 4500 Oak Street, Vancouver, BC, V6H 3N1, Canada; 4 Dept. of Pathology and Laboratory Medicine, BC Children's Hospital,4480 Oak Street, Vancouver, BC, V6H 3N1, Canada; 5 Genome British Columbia, 500-555 West 8th Avenue, Vancouver, BC, V5Z 1C6, Canada

## Abstract

**Background:**

Genomic deletions and duplications are important in the pathogenesis of diseases, such as cancer and mental retardation, and have recently been shown to occur frequently in unaffected individuals as polymorphisms. Affymetrix GeneChip whole genome sampling analysis (WGSA) combined with 100 K single nucleotide polymorphism (SNP) genotyping arrays is one of several microarray-based approaches that are now being used to detect such structural genomic changes. The popularity of this technology and its associated open source data format have resulted in the development of an increasing number of software packages for the analysis of copy number changes using these SNP arrays.

**Results:**

We evaluated four publicly available software packages for high throughput copy number analysis using synthetic and empirical 100 K SNP array data sets, the latter obtained from 107 mental retardation (MR) patients and their unaffected parents and siblings. We evaluated the software with regards to overall suitability for high-throughput 100 K SNP array data analysis, as well as effectiveness of normalization, scaling with various reference sets and feature extraction, as well as true and false positive rates of genomic copy number variant (CNV) detection.

**Conclusion:**

We observed considerable variation among the numbers and types of candidate CNVs detected by different analysis approaches, and found that multiple programs were needed to find all real aberrations in our test set. The frequency of false positive deletions was substantial, but could be greatly reduced by using the SNP genotype information to confirm loss of heterozygosity.

## Background

Chromosomal abnormalities frequently contribute to human disorders including cancer [1-3] and mental retardation (MR) [4-6], and characterization of these DNA alterations is important for both diagnosis and understanding of disease mechanisms. A surprising recent finding has been the extent to which genomic copy number variants (CNVs) also exist in the normal population [7-13]. Such variation may represent an important class of mutations that predispose to disease.

Conventional cytogenetic studies such as karyotyping are routinely used to detect genomic deletions and duplications involving more than 5–10 Mb, but detection of submicroscopic aberrations requires higher resolution approaches. Oligonucleotide microarray technologies offer high resolution, scalable methods for whole genome screening and can detect previously unidentified CNVs [6,14-17]. Among these approaches, the Affymetrix GeneChip^® ^Mapping Assay [18,19] is increasingly used for detecting CNVs in human DNA. This method involves a whole genome sampling analysis (WGSA) combined with high-density SNP genotyping oligonucleotide arrays. The first such arrays contained 1,494 SNPs, and the subsequent 10 K arrays consisted of 11,555 SNPs [14]. Further development resulted in the 100 K array set with probes for 116,204 SNPs [16], and now the 500 K array set containing 500,568 SNPs [18] is available. All these arrays can be used to estimate copy number changes from probe intensities, determine SNP genotypes by allele-specific hybridization, confirm loss of heterozygosity, detect uniparental disomy, identify non-paternity and determine haplotypes and parental origin of CNVs.

A number of software packages are available for analysis of oligonucleotide arrays [14,20-23]. Three software packages, listed in Table 1, are currently in common use for copy number analysis of Affymetrix 100 K SNP WGSA data: Copy Number Analyser for GeneChip^® ^arrays (CNAG) [22,24], DNA-Chip Analyzer (dChip) [23,25] and Affymetrix GeneChip^® ^Chromosome Copy Number Analysis Tool (CNAT) [14,18]. All of these software packages perform normalization, scaling and feature extraction of signal intensities, and enable detection of copy number alterations, but each package uses a different algorithm for these functions. Briefly, CNAG normalizes and scales the test sample against a "best-fit" user-defined reference set and corrects the signal intensity ratios for the differences in PCR product length and GC content. After feature extraction a Hidden Markov Model (HMM) algorithm is applied to infer copy numbers along each chromosome [22]. dChip normalizes and scales data within and between chips using a procedure established for Affymetrix GeneChip^® ^arrays [23], and then compares the test sample to a user-defined reference set of samples to estimate copy numbers in the test sample. This output is then used by an HMM to infer copy numbers [23]. CNAT compares a test sample to a reference set of 106 samples provided by Affymetrix [18] or to a user-defined reference set to estimate the copy number of each SNP locus, and then applies a Kernel Smoothing algorithm to identify the regions of copy number alteration [14]. The relative performance of these methods in performing high throughput oligonucleotide array normalization, scaling and feature extraction and their performance in the sensitivity or specificity of CNV detection have not previously been reported, nor have the effects of different reference sets on CNV discovery. Accordingly, in this study we compared the performance of CNAG, dChip and CNAT software (Table 1) using synthetic data and an empirical data set that contains CNVs validated predominantly by fluorescent *in situ *hybridization (FISH). We report assessment of the normalization, scaling and feature extraction algorithms of these packages, as well as assessment of the approaches used for identification of CNVs and their boundaries. In addition, we tested the impact of reference set size and composition on CNV detection with each software package. Finally, we estimated the true and false positive detection rates of these various approaches for the identification of genomic gains or losses.

**Table 1:** List of copy number analysis software packages evaluated

**Developed for Affymetrix GeneChip Mapping 100 K Array Data Analysis**					
			
**Software**	**Name**	**Normalization, scaling and feature extraction^a^**	**Smoothing**	**Estimation**	**Reference**
CNAG 1.1	Copy Number Analyser for GeneChip	yes	yes	yes	[22]
dChip (Nov 17, 2005)	DNA-Chip Analyzer	yes	yes	yes	[23]
CNAT 3.0	Chromosome Copy Number Analysis Tool	yes	yes	no	[14]
					
**Developed for Array CGH Data Analysis**					
			
**Software**	**Name**	**Normalization, scaling and feature extraction**	**Smoothing**	**Estimation**	**Reference**

GLAD (R)	Gain and Loss Analysis of DNA	no	yes	yes	[26]

^a^Capability to perform normalization, scaling and feature extraction on Affymetrix GeneChip^® ^Mapping 100 K array data.

## Results and discussion

The purpose of this study was to compare the performance of various software packages and the effect of different reference sets on identification of CNVs in Affymetrix 100 K SNP array data. We performed the evaluations described here using a synthetic data set and an empirical data set that we generated from 331 individuals (Additional file 1). The sample set was derived from 107 patients with mental retardation (MR) and their unaffected mothers and fathers, as well as 10 unaffected siblings of the patients. Several of the individuals studied have CNVs that were validated using independent methods [6].

**Figure 1:**
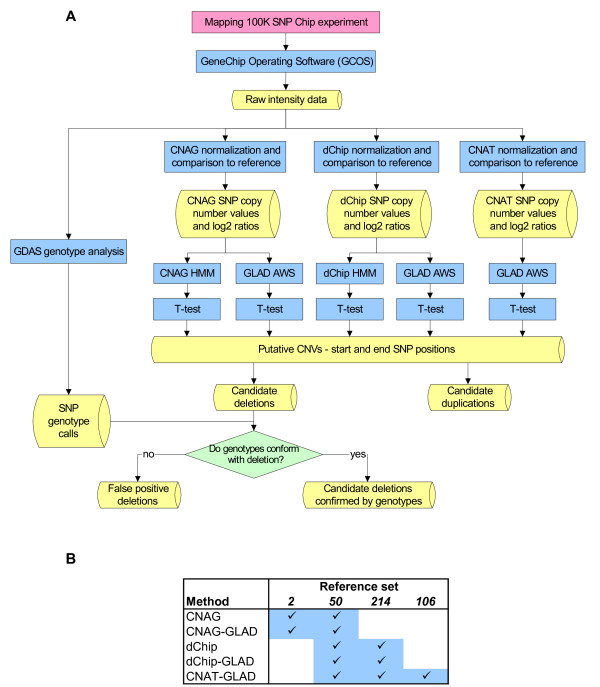
Overview of the data analysis process. **A) **Methods appear in blue, and data in yellow. **B) **The reference sets used for each analysis method are as follows. '***2***': within each MR trio (child, mother and father), three comparisons were done – child to father as reference, child to mother as reference, and father to mother as reference. '***50***': each sample was compared to a reference set of 50 unaffected mothers of children with MR. These 50 mothers selected for this reference set had the lowest numbers of CNVs detected by dChip compared to other mothers. '***214***': each sample was compared to a reference set that included all 214 unaffected parents (107 mothers and 107 fathers) of the children with MR. '***106***': a default reference set of 106 individuals provided by Affymetrix for copy number analysis with CNAT [18].

We performed 100 K SNP WGSA experiments using 662 arrays of which 331 were Xba 50 K chips and 331 were Hind 50 K chips (Additional file 1). From individual oligonucleotide probe intensities, we determined the SNP genotypes (Figure 1; Methods) and performed initial copy number analysis using each of the software packages listed in Table 1. Of the software packages we analyzed, only those developed for Affymetrix GeneChip Mapping 100 K arrays are capable of normalization, scaling and feature extraction of Affymetrix data (Table 1). Hence, we used CNAG, dChip or CNAT to perform this procedure on our array data.

CNAG and dChip use HMM-based algorithms to detect regions of genomic gains and losses and estimate their boundaries (Table 1). CNAT provides plots of copy number and associated p-values along each chromosome, but does not report CNVs or their boundaries. For the estimation of CNVs and their breakpoints, we evaluated the utility of CNAG, dChip and GLAD [26], the latter developed originally for array-CGH data analysis (Table 1).

### Detection of candidate copy number variants from synthetic data

As an initial assessment of the software packages, we constructed a synthetic data set in which we purposely introduced artificial CNVs, and then measured CNV detection performance, including true and false positive detection rates, of the software approaches.

Our data set contained 30 artificial normalized array results produced from a normal individual's genome and subsequent comparison to a reference set of 50 individuals (Methods). Normalization was performed using CNAG, dChip and CNAT for these synthetic array results (10 by each software). We then introduced 100 simulated CNVs into each of the 30 synthetic samples with probe set widths ranging from 5 to 23 and copy numbers ranging from 0.3 to 3.0 (Methods). Detection of CNVs in these normalized data was then performed using dChip and GLAD (Methods). CNV detection could not be performed using CNAG, because this software does not accept intermediate stage normalized data as input.

The total numbers of putative CNVs detected from the synthetic arrays, and assessments of false positive and false negative rates are shown in Table 2. None of the software detected all true CNVs as the true positive rates fell between 0.23 (CNAT-GLAD, Hind data, Table 2) and 0.58 (CNAG-GLAD, Xba data, Table 2). All of the approaches had false discovery rates ranging between 0 (CNAG-GLAD, Hind data, Table 2) and 0.44 (dChip, Xba data, Table 2). We observed generally superior performance in the detection of deletions compared to the detection of duplications. We found that dChip analysis of the synthetic data resulted in the identification of the largest number of putative CNVs but yielded fairly low true positive rates (0.32 and 0.26 for Xba and Hind data, respectively) and the highest false discovery rates (0.44 and 0.42 for Xba and Hind data, respectively) (Table 2). In this analysis the CNAG-GLAD approach showed the best overall true positive rates (0.58 and 0.42 for Xba and Hind data, respectively) and lowest false discovery rates (0.009 and 0 for Xba and Hind data, respectively).

**Table 2:** Candidate copy number variants from synthetic data

**Total from Xba data**										
							
**Method^a^**	**# CNVs**	**# Duplications**	***%***	**#Deletions**	***%***	**# True CNVs**	**# Unique True CNVs^b^**	**True Positive Rate (power)^c^**	**# False Positive CNVs**	**False Discovery Rate**
CNAG-GLAD	334	20	*6*	314	*94*	331	58	*0.58*	3	*0.009*
dChip	381	166	*44*	215	*56*	213	32	*0.32*	168	*0.44*
dChip-GLAD	70	0	*0*	70	*100*	70	31	*0.31*	0	*0*
CNAT-GLAD	111	10	*9*	101	*90*	101	36	*0.36*	10	*0.09*
										
**Total from Hind data**										
							
**Method**	**# CNVs**	**# Duplications**	***%***	**#Deletions**	***%***	**# True CNVs**	**# Unique True CNVs**	**True Positive Rate (Power)**	**# False Positive CNVs**	**False Discovery Rate**

CNAG-GLAD	70	11	*16*	59	*84*	70	42	*0.42*	0	*0*
dChip	269	91	*34*	178	*66*	155	26	*0.26*	114	*0.42*
dChip-GLAD	101	5	*5*	96	*95*	94	33	*0.33*	7	*0.07*
CNAT-GLAD	49	0	*0*	49	*100*	48	23	*0.23*	1	*0.02*

^a^Where two software packages are listed, the first one was used for normalization and the second for CNV detection.

^b^CNVs with different chromosomal locations and breakpoints.

^c^The number of true (synthetic) CNVs per array is 100.

### Detection of candidate copy number variants from empirical data

To assess the performance of the software approaches on empirical data, we next analyzed 662 Affymetrix SNP arrays, employing five approaches that in total used four software packages (Figure 1, Table 3). To detect regions of genomic gains and losses after normalization, scaling and feature extraction by CNAG, dChip or CNAT, we applied the HMM algorithms of CNAG and dChip, as well as the adaptive weights smoothing (AWS) algorithm of GLAD (Figure 1, Table 3). Due to the difference in normal X chromosome copy numbers between males and females, detection of X chromosome CNVs requires more complex approaches than autosomal CNVs, and not all of the software packages tested here were able to score genomic copy number along the sex chromosomes. Hence, we focused on copy number assessment of autosomal regions. To identify a candidate CNV, we arbitrarily imposed a requirement for at least four adjacent SNPs that demonstrated a similar apparent gain or loss of copy number.

**Table 3:** Candidate copy number variants from empirical data

**All from Xba data**								
					
**Method**	**Reference Set**	**#CNVs**	**# Duplications**	***%*^a^**	**# Deletions**	***%*^a^**	**# False Positive Deletions^b^**	***%*^c^**
1-CNAG	2	3,210	1,755	*55*	1,455	*45*	970	*67*
2-CNAG	50	924	820	*89*	104	*11*	35	*34*
3-CNAG-GLAD	2	1,850	996	*54*	854	*46*	343	*40*
4-CNAG-GLAD	50	340	69	*20*	271	*80*	62	*23*
5-dChip	50	31,354	19,093	*61*	12,261	*39*	3,830	*31*
6-dChip	214	5,443	4,076	*75*	1,367	*25*	452	*33*
7-dChip-GLAD	50	1,292	66	*5*	1,226	*95*	456	*37*
8-dChip-GLAD	214	1,207	30	*2*	1,177	*98*	402	*34*
9-CNAT-GLAD	50	701	253	*36*	448	*64*	214	*48*
10-CNAT-GLAD	106	454	232	*51*	222	*49*	98	*44*
11-CNAT-GLAD	214	866	240	*28*	626	*72*	363	*58*
								
**p <= 0.05 and copy number < 1.25 or > 2.75 from Xba data**								
					
**Method**	**Reference Set**	**#CNVs**	**# Duplications**	***%***	**# Deletions**	***%***	**# False Positive Deletions**	***%***

1-CNAG	2	444	361	*81*	83	*19*	21	*25*
2-CNAG	50	235	211	*90*	24	*10*	3	*13*
3-CNAG-GLAD	2	416	332	*80*	84	*20*	27	*32*
4-CNAG-GLAD	50	133	48	*36*	85	*64*	17	*20*
5-dChip	50	17,034	4,846	*28*	12,188	*72*	3,804	*31*
6-dChip	214	2,273	907	*40*	1,366	*60*	452	*33*
7-dChip-GLAD	50	1,042	27	*3*	1,015	*97*	313	*31*
8-dChip-GLAD	214	1,027	22	*2*	1,005	*98*	283	*28*
9-CNAT-GLAD	50	426	87	*20*	339	*80*	115	*34*
10-CNAT-GLAD	106	272	88	*32*	184	*68*	61	*33*
11-CNAT-GLAD	214	540	117	*22*	423	*78*	172	*41*
								
**All from Hind data**								
					
**Method**	**Reference Set**	**#CNVs**	**# Duplications**	***%***	**# Deletions**	***%***	**# False Positive Deletions**	***%***

1-CNAG	2	2,127	1,161	*55*	966	*45*	638	*66*
2-CNAG	50	324	202	*62*	122	*38*	41	*34*
3-CNAG-GLAD	2	1,299	697	*54*	602	*46*	206	*34*
4-CNAG-GLAD	50	366	20	*5*	346	*95*	87	*25*
5-dChip	50	21,124	17,843	*84*	3,281	*16*	1,402	*43*
6-dChip	214	5,792	4,603	*79*	1,189	*21*	469	*39*
7-dChip-GLAD	50	790	42	*5*	748	*95*	253	*34*
8-dChip-GLAD	214	806	41	*5*	765	*95*	274	*36*
9-CNAT-GLAD	50	650	108	*17*	542	*83*	210	*39*
10-CNAT-GLAD	106	360	90	*25*	270	*75*	108	*40*
11-CNAT-GLAD	214	462	56	*12*	406	*88*	161	*40*
								
**p <= 0.05 and copy number < 1.25 or > 2.75 from Hind data**								
					
**Method**	**Reference Set**	**#CNVs**	**# Duplications**	***%***	**# Deletions**	***%***	**# False Positive Deletions**	***%***

1-CNAG	2	377	300	*80*	77	*20*	26	*34*
2-CNAG	50	52	12	*23*	40	*77*	15	*38*
3-CNAG-GLAD	2	383	287	*75*	96	*25*	29	*30*
4-CNAG-GLAD	50	140	9	*6*	131	*94*	38	*29*
5-dChip	50	6,488	3,230	*50*	3,258	*50*	1,392	*43*
6-dChip	214	1,822	637	*35*	1,185	*65*	468	*39*
7-dChip-GLAD	50	744	23	*3*	721	*97*	238	*33*
8-dChip-GLAD	214	748	18	*2*	730	*98*	245	*34*
9-CNAT-GLAD	50	547	52	*10*	495	*90*	182	*37*
10-CNAT-GLAD	106	311	67	*22*	244	*78*	89	*36*
11-CNAT-GLAD	214	426	48	*11*	378	*89*	150	*40*

^a^Percentage of all candidate CNVs.

^b^Numbers of false positive deletions were estimated using SNP genotype data (Methods).

^c^This is the percentage of false positives among the deletions (# false positive deletions/# deletions * 100).

To determine the effect of reference set size and composition on CNV detection, we used four reference sets in our analyses (Figure 1, Table 3). The algorithmic differences between CNAT, dChip and CNAG impose different requirements with regards to the size range of the reference set used. However, by default, all of the software packages we used assume that the reference set has a mean copy number of 2.0 at all autosomal locations. A large reference set would usually satisfy this assumption because, in such a set, rare polymorphic CNVs will have negligible effects. A large reference set also provides the advantage of reducing noise arising from the comparison. However, common polymorphic CNVs in the reference sets could still affect the results.

Pair wise comparisons of one sample to another can only be performed with CNAG [22]. This may be useful in the case of parent-offspring "trio" analyses, such as reported in [6]. Direct comparison of array data derived from a child to data derived from the parents is the most straight-forward means of distinguishing *de novo *mutations from inherited CNVs, and the boundaries of inherited aberrations should usually be the same in the parent and child. Thus, we tested CNAG, as well as CNAG normalization, scaling and feature extraction combined with GLAD CNV detection (CNAG-GLAD) using three pair-wise comparisons within each trio – child to father, child to mother, and father to mother (Figure 1, Table 3, Methods). We refer to this analysis within trios as a "reference set of 2".

dChip and CNAT require the use of larger reference sets: the minimum sizes required are 10 for dChip [23] and 50 for CNAT [18]. So that one consistent reference set could be used to compare the performance of all three software packages, we chose a reference set of 50 unaffected mothers of children with MR (Figure 1, Table 3, Methods). These 50 mothers chosen were those with the fewest candidate CNVs identified by dChip, compared to the other mothers in our data set (using a reference set of all 214 parents). With dChip and CNAT, it is possible to increase the size of the reference set further, so we tested whether this would be advantageous. For this purpose, we assembled a reference set of 214 unaffected parents of children with MR (Figure 1, Table 3, Methods). For CNAT, there is a default reference set of 106 individuals provided by Affymetrix, which was also evaluated (Figure 1, Table 3, Methods).

The lists of candidate CNVs and their boundaries identified in the 331 samples by CNAG using reference sets of 2 and 50 individuals are shown in Additional files 2 and 3, respectively. The CNVs detected with the CNAG-GLAD approach are listed in Additional file 4 (reference set of 2) and Additional file 5 (reference set of 50). Putative CNVs found with dChip are shown in Additional files 6 and 7 (reference set of 50) and Additional file 8 (reference set of 214). CNVs detected by GLAD from the feature extracted data by dChip (dChip-GLAD) are shown in Additional file 9 (reference set of 50) and Additional file 10 (reference set of 214). Candidate CNVs identified using GLAD from SNP copy number log_2_-ratios calculated by CNAT (CNAT-GLAD) are listed in Additional file 11 (reference set of 50), Additional file 12 (Affymetrix default reference set of 106) and Additional file 13 (reference set of 214).

Table 3 summarizes the numbers of candidate genomic deletions and duplications identified using each of these combinations of methods and reference sets on the 331 individuals studied. The data are presented for the Xba and Hind arrays separately, so a CNV that was identified in a particular sample by both array types is listed under both (Table 3). There is great variability in the numbers and types of CNVs detected from the same sample set with different analysis approaches. The fewest candidate CNVs were detected using CNAG-GLAD and CNAG with the reference set of 50 – 340 from Xba and 324 from Hind data, respectively (Table 3). The most putative CNVs were identified by dChip with the reference set of 50 – 31,354 from Xba and 21,124 from Hind data (Table 3).

The types of candidate CNVs detected also varied greatly among the 11 approaches. Duplications accounted for between 2% and 89% of all CNVs (Table 3). The lowest proportion of duplications, and thus the highest proportion of deletions, was identified by dChip-GLAD. The three highest proportions of duplications and lowest proportions of deletions were detected by dChip with the reference sets of 50 and 214, and by CNAG with the reference set of 50 (Table 3).

For three Hind and two Xba arrays, each from a different sample, candidate CNVs were not detected by any approach. Data from 97 Hind and 90 Xba chips predicted 30 or more putative CNVs by at least one method. However, none of the arrays had 30 or more aberrations detected by all of the 11 approaches.

### False positive rate

The ultimate approach to determining the false positive rate of each copy number analysis method would be to attempt validation of each candidate CNV using an independent method. A subset of putative CNVs was confirmed using FISH (Table 4) [6], but it was not feasible to do this for all of the many thousands of candidate CNVs detected in this study (Table 3).

**Table 4:** Detection of validated CNVs

**Sample ID**	**CNV**	**Chr**	**Length (kb)**	**CNAG Ref2^a^**	**CNAG Ref50**	**CNAG-GLAD Ref2**	**CNAG-GLAD Ref50**	**dChip Ref50**	**dChip Ref214**	**dChip-GLAD Ref50**	**dChip-GLAD Ref214**	**CNAT-GLAD Ref50**	**CNAT-GLAD Ref106**	**CNAT-GLAD Ref214**	**# Methods detected**	***% Methods detected***
3476c	del^b^	4	10,655	1	1	1	1	1	1	1	1	1	1	1	11	*100*
1895c	del	13	4,887	1	1	1	1	1	1	1	1	1	1	1	11	*100*
4818c	del	12	3,204	1	1	1	1	1	1	1	1	1	1	1	11	*100*
9143c	del	11	3,175	1	1	1	1	1	1	1	1	1	1	1	11	*100*
8326c	del	14	1,923	1	1	1	1	1	1	1	1	1	1	1	11	*100*
6235c	del	10	1,737	1	1	1	1	1	1	0	1	0	1	1	9	*82*
6545c	del	7	785	1	1	1	1	1	1	1	1	1	0	1	10	*91*
7807c	del	22	731	1	1	1	1	1	1	1	1	1	1	1	11	*100*
4357c	del	6	595	1	1	1	1	1	1	1	1	1	1	1	11	*100*
4357m	del	6	595	1	1	1	1	1	1	1	1	1	1	1	11	*100*
4357c	del	6	353	1	1	1	1	1	1	1	1	1	1	1	11	*100*
4357m	del	6	353	1	1	1	1	1	1	1	1	1	1	1	11	*100*
5003c	del	2	294	1	1	1	1	0	0	1	1	1	1	1	9	*82*
7551c	del	2	220	1	1	1	1	1	1	1	1	1	1	1	11	*100*
7551m	del	2	220	1	1	1	0	1	1	1	1	1	1	1	10	*91*
1280c	del	4	192	1	1	1	1	1	0	1	1	1	0	1	9	*82*
1280m	del	4	192	1	1	1	1	1	0	1	1	1	0	1	9	*82*
0674c	del	2	147	1	1	1	1	0	0	1	1	1	1	1	9	*82*
0674f	del	2	147	1	0	1	1	1	0	1	1	1	0	1	8	*73*
5566c	del	14	130	0	0	1	1	0	0	0	0	1	0	1	4	*36*
6789c	del	14	68	1	0	1	1	0	0	0	0	1	0	1	5	*45*
6789m	del	14	68	1	0	1	1	0	0	0	0	0	0	1	4	*36*
3476c	del	1	66	1	0	1	1	1	0	1	1	1	0	1	8	*73*
3476m	del	1	66	1	0	1	0	0	0	1	1	1	0	1	6	*55*
6607c	del	20	57	0	0	1	0	0	0	0	0	0	0	0	1	*9*
6607m	del	20	57	0	0	1	0	0	0	0	0	0	0	0	1	*9*
8785c	del	18	43	0	0	0	1	1	0	1	1	1	1	1	7	*64*
8785f	del	18	43	0	0	0	0	1	0	1	1	0	0	0	3	*27*
9299f	del	9	38	0	0	1	1	0	0	1	1	1	0	1	6	*55*
9299c	del	9	38	0	0	1	1	0	0	1	0	1	0	1	5	*45*
8379c	dup	10	23,842	1	1	1	1	1	1	1	1	1	1	1	11	*100*
4794c	dup	16	3,356	1	1	1	0	1	1	0	0	0	0	0	5	*45*
8379c	dup	15	1,481	1	1	1	1	1	1	1	1	1	0	1	10	*91*
3595c	dup	15	781	1	1	1	0	1	1	0	0	1	0	1	7	*64*
3595m	dup	15	781	0	1	0	0	0	0	0	0	0	0	1	2	*18*
3923c	dup	11	494	1	1	0	1	1	1	0	0	1	0	1	7	*64*
3923m	dup	11	494	0	0	0	0	1	1	0	0	0	0	0	2	*18*
6168c	dup	17	324	1	0	1	0	1	1	0	0	0	0	0	4	*36*
																
**Number of validated CNVs detected**				29	24	33	28	27	21	26	26	29	17	32		
***% of validated CNVs detected***				*76*	*63*	*87*	*74*	*71*	*55*	*68*	*68*	*76*	*45*	*84*		

^a^Detection of CNV from at least one type of array data (Xba or Hind or both). 1 means detected, 0 means not detected.

^b^The method of validation for each CNV is shown in Additional file 15.

To obtain an estimate of false positive rates among a larger number of candidate deletions, we used the SNP genotype data, assuming that deletions (with copy number of 1 or 0) should not contain heterozygous genotype calls (Figure 1, Methods). The average proportion of false positive deletions identified by SNP heterozygosity was 40%, ranging from 23% to 67% in the Xba data, and between 25% and 66% in the Hind data (Table 3). In both array types, the CNAG-GLAD combination with the reference set of 50 exhibited the lowest false positive deletion rate, and CNAG with a reference set of size 2 produced the highest. We note that these false positive rates are likely underestimates, especially for short CNVs, because stretches of homozygous SNPs could also occur by chance in regions with normal copy number.

The software packages tested here apply different algorithms and statistics for CNV detection. We examined the distribution of SNP copy numbers and found that they showed the characteristics of a Gaussian distribution. Thus, to assess and compare the significance of CNVs detected by these different approaches, we performed a t-test as follows. First, we calculated the log_2_-ratios of test sample copy numbers versus reference copy numbers for each SNP. Next we calculated the mean and standard deviation (SD) of these log_2_-ratios within each candidate CNV, and also for the rest of the same chromosome excluding the region affected by the CNV. We then compared these values using a t-test, and obtained the corresponding p-values (Additional files 2,  3, 4, 5, 6, 7, 8, 9, 10, 11, 12, 13). We then filtered the candidate CNVs using an uncorrected p <= 0.05 cutoff along with arbitrary copy number value thresholds of <1.25 for deletions and >2.75 for duplications. The candidate CNVs that passed these thresholds are summarized in Table 3. As expected, application of these cutoff values resulted in fewer CNVs and also reduced the false positive deletion rates in most instances. However, the false positive deletion call rates remained substantial, averaging 32%. Lower p-value thresholds further reduced the numbers of candidate CNVs, as expected (not shown). However, substantial rates of false positive deletions still remained even with a p <= 0.00001 cutoff, with an average false positive deletion rate of 28%.

**Figure 2:**
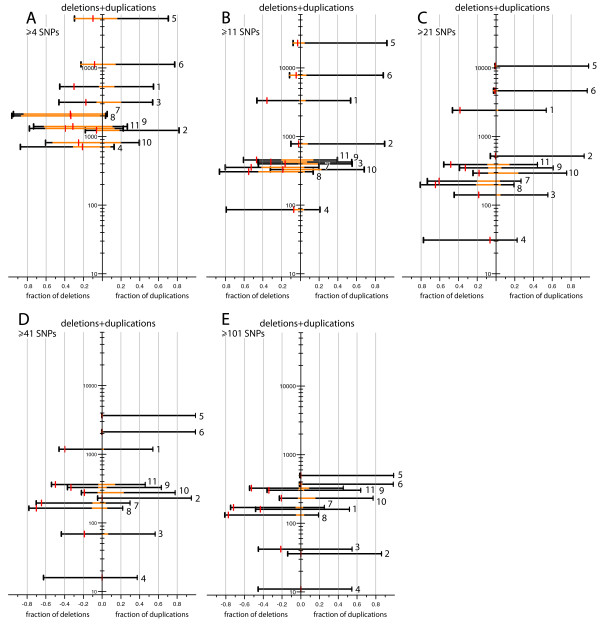
Size distribution of candidate CNVs detected. The five plots show numbers of candidate copy number gains and losses identified using Xba and Hind arrays, arranged according to the numbers of SNPs within the aberrations: **A) **all CNVs (>= 4 SNPs); **B) **CNVs >= 11 SNPs; **C) **CNVs >= 21 SNPs; **D) **CNVs >= 41 SNPs and **E) **CNVs >= 101 SNPs. The y-axis value of each horizontal line represents the total number of CNVs detected by a given method: **1 **– CNAG Ref2; **2 **– CNAG Ref50; **3 **– CNAG-GLAD Ref2; **4 **– CNAG-GLAD Ref50; **5 **– dChip Ref50; **6 **– dChip Ref214; **7 **– dChip-GLAD Ref50; **8 **– dChip-GLAD Ref214; **9 **– CNAT-GLAD Ref50; **10 **– CNAT-GLAD Ref106; **11 **– CNAT-GLAD Ref214 (the reference sets are described in Figure 1 and in the Methods.) The left and right side of each panel correspond to the fraction of deletions and duplications, respectively. The orange bars within the black lines show the fraction of CNVs that passed the following confidence thresholds: p <= 0.05 (t-test) and copy number < 1.25 for deletions (left); or p <= 0.05 (t-test) and copy number > 2.75 for duplications (right). The fractions of false positive deletion calls, calculated based on SNP heterozygosity, are indicated by the red vertical bars on the left side of each panel. For example, the y-axis value of the top line (5) in plot 'A' indicates the total number of candidate CNVs (52,478) including at least 4 consecutive SNPs identified by dChip Ref50 (from Xba and Hind data). 30% of the 52,478 putative CNVs were deletions (left) and 70% were duplications (right). 99% of the deletions (orange fraction of the line, left) and 22% of the duplications (orange fraction of the line, right) passed our p-value <= 0.05 and copy number (<1.25 or >2.75) thresholds described above. 34% of the candidate deletions were considered to be false positives, indicated by the red bar (left).

To assess how detection and false positive rates were affected by the number of SNPs included in candidate CNVs, we counted the number of candidates that included at least 4, 11, 21, 41 or 101 SNPs (Figure 2). We also calculated false positive deletion rates on the basis of SNP heterozygosity at each level and applied p-value and copy number thresholds as described above (Figure 2). We note that the rate of false positive deletion calls in the smallest size class (4–10 SNPs) may be unrealistically low in our analysis, because homozygosity is more likely to occur by chance over a few adjacent SNPs than over many. However, there was a high number of CNVs that passed our p-value (p <= 0.05) and copy number (<1.25 or >2.75) thresholds and that were predicted by <= 10 SNPs, indicating that many of the putative CNVs in this small size range may be real. Interestingly, the false positive call rate was often relatively high and the percentage of CNVs that passed the p-value and copy number thresholds was often relatively low in the largest CNV size class (>= 101 SNPs) compared to the other categories (Figure 2). The majority of false positive CNVs in this size range exhibited copy numbers that averaged only a little more or less than 2.0, but the change may have appeared significant because of the large number of SNPs involved.

### Candidate CNVs predicted by multiple methods

Putative CNV regions identified by at least two software/reference set combinations from Xba or Hind data or both from the same sample are presented in Additional file 14. These regions were predicted by the software platforms without applying additional filters based on p-value or copy number thresholds. Because distinct approaches and the two different chips in the 100 K set often detect slightly different boundaries for a particular CNV, we defined mutually predicted CNVs in this analysis as those sharing at least 50% of the base pairs within a genomic segment defined by the SNPs included in the deletion or duplication.

Mutually predicted candidate CNVs consisting of fewer than 50 consecutive SNPs are listed in Additional file 14A. In this size range, a total of 8,649 putative CNVs consisting of 5,418 duplications (63%) and 3,231 deletions (37%) were detected in our sample set of 331 individuals using two or more methods. 7,497 (86%) of these putative 8,649 CNVs (<50 SNPs) were detected by 2 distinct software/reference set combinations, 919 (11%) by 3 or 4, and 233 (3%) by 5 or more approaches. 1,512 of the candidate deletions predicted by more than one approach (47% of 3,231) were considered to be false positive calls on the basis of SNP heterozygosity (Methods, Figure 1).

Mutually predicted putative CNVs of 50 or more consecutive SNPs are listed in Additional file 14B. A total of 1,084 such candidate CNVs were identified by at least two methods, including 926 duplications (85%) and 158 deletions (15%). Of these larger CNVs, 963 (89%) were identified by 2 distinct software/reference set combinations, 106 (10%) by 3 or 4, and 15 (1%) via 5 or more approaches. 154 (97% of 158) deletions predicted by more than one approach were considered to be false positive calls on the basis of SNP heterozygosity (Methods). We validated 3 of the remaining candidate deletions using FISH (Additional file 14, Table 4); the fourth one was not tested.

### Rate of detection of confirmed CNVs

To determine the detection rate of real CNVs by each of the software/reference set combinations, we assessed 38 CNVs (30 deletions and 8 duplications) that were confirmed by independent experimental approaches (Table 4, Additional file 15) [6]. Some of these were inherited CNVs that had been demonstrated in both the child and a parent of one MR family. Other confirmed CNVs occurred *de novo *in a child with MR and were shown not to be present in either parent. SNP genotypes were used to confirm paternity in all cases.

The confirmed deletions in this set all had a copy number of 1 in the involved genomic region, and the confirmed duplications all had a copy number of 3. Deletions of ~200 kb or larger were identified by most or all (between 9 and 11) of the 11 software/reference set combinations used. As expected, detection rates were lower for smaller deletions (Table 4). Successful detection of duplications had lower rates overall (Table 4). Surprisingly, a rather large 3.3 Mb duplication was detected by only 5 of the 11 software/reference set combinations (Table 4, Additional file 15). Within this genomic segment, the average distance between SNPs was ~280 kb (Additional file 15), which is substantially greater than the 23.6 kb average distance between SNPs across the whole genome in the 100 K array set [16].

No single method identified all 38 of the confirmed CNVs. CNAG-GLAD with the reference set of 2 and CNAT-GLAD with the reference set of 214 had the highest rates for detection, identifying 33 and 32 of the 38 confirmed CNVs, respectively (Table 4). Two large deletions were divided by dChip and the CNAG-GLAD combined approach into multiple smaller deletions (2–4 each), instead of the single CNVs predicted by alternate approaches (Additional file 15).

### Candidate CNVs per individual

To estimate the average number of CNVs per genome in our sample set, we chose a combination of three copy number analysis approaches that resulted in optimal true positive detection rate: CNAG-GLAD with the reference set of 2, dChip with the reference set of 50 and CNAT-GLAD with the reference set of 214. These three methods together detected all of the 38 confirmed CNVs in this study (Table 4, Additional file 15). We generated a list of candidate CNVs that were identified by at least one of these three approaches, from at least one array (Xba or Hind). To reduce the false positive detection rate, we eliminated all of the putative aberrations that did not meet the following criteria: p <= 0.05 (t-test); and copy number <= 1.25 (for deletions) or >= 2.75 (duplications). Deletions considered false positive based on SNP heterozygosity were also eliminated. We then calculated the average numbers of remaining candidate CNVs per individual in the 107 children with MR, and in the 224 unaffected parents and siblings of the affected children.

In the 224 unaffected individuals we found an average of 39 candidate CNVs per genome, consisting of 20 deletions and 19 duplications. The average size of the deletions was 157 kb (in the range between 190 bp and 5.5 Mb), and the average size of the duplications was 244 kb (ranging between 115 bp and 16.7 Mb). In the affected children, the average number of candidate CNVs was 45, including 26 deletions and 19 duplications. The average size of the deletions was 191 kb (ranging between 220 bp and 11.3 Mb), and the average size of the duplications was 208 kb (ranging between 220 bp and 23.8 Mb).

### Theoretical resolving power

**Figure 3:**
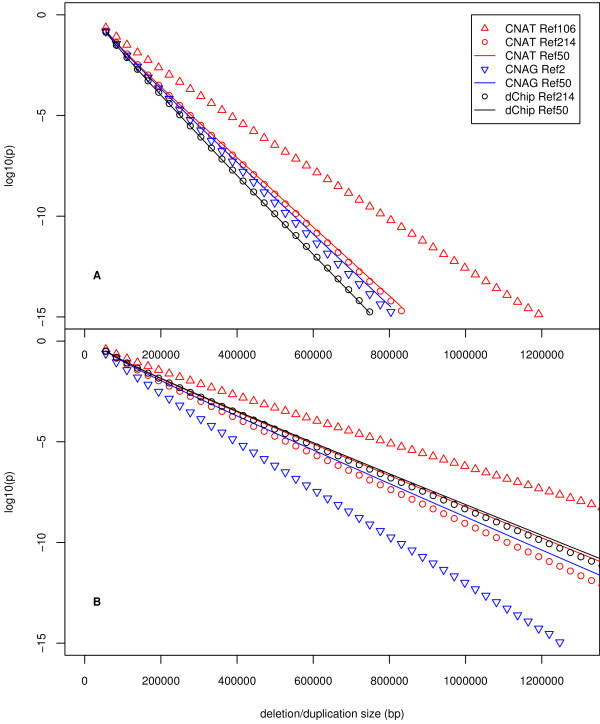
Theoretical resolving power of CNAG, dChip and CNAT with reference sets of 2, 50, 106 and 214 (see Methods and Figure 1 legend). The resolving power was defined as the average size of the smallest one-copy deletion or duplication that could be detected with a given method at a given confidence level. The theoretical p-value (in log_10 _scale) is shown as a function of the deletion **(A) **or duplication **(B) **size detected from Affymetrix GeneChip 100 K Xba and Hind data. For a given p-value, e.g. 10^-5^, the theoretical minimum size of detectable deletion or duplication is shown for each method. For a deletion or duplication of a given size, e.g. 400,000 bp, the theoretical p-values are shown for each method.

The ability to estimate genomic gains and losses and to define their boundaries is dependent on the normalization, scaling and feature extraction of the raw intensity data. More effective normalization and feature extraction yields higher signal-to-noise ratios, which enable superior detection of regions with altered copy numbers. Using SNP copy number data from the 30 validated deletions and 8 confirmed duplications listed in Table 4, we calculated theoretical resolving powers of the normalization, scaling and feature extraction algorithms used by CNAG, dChip and CNAT with the various reference sets described above (Figure 1B, Methods). We defined the resolving power as the average size of the smallest single copy deletion or duplication that could be detected at a given confidence level. Mean test versus reference SNP copy number log_2_-ratios were calculated from the data following feature extraction, and they showed the characteristics of a Gaussian distribution. The Welch t-test was then computed to compare mean SNP copy number ratios within a given CNV against the rest of the chromosome (Methods). For this calculation, we assumed that SNPs were uniformly distributed throughout the genome. We then estimated the p-values that would be obtained for hemizygous deletions (copy number 1) and single copy duplications (copy number 3) containing increasing numbers of adjacent SNPs using the means and standard deviations obtained from 30 confirmed deletions and 8 confirmed duplications (Methods). In genomic regions where the SNP density is higher or lower than average, corresponding p-values would be lower or higher than those presented in Figure 3, but variation in SNP density would affect the p-values across all of the methods similarly. Therefore, even though absolute p-values change with SNP density, the relative p-values presented here provide a valid comparison.

The resolving power calculated from the weighted average of the 30 validated deletions (Table 4) is shown in Figure 3A. The resolving power calculated from the weighted average of the 8 confirmed duplications (Table 4) is shown in Figure 3B. We observed that the Affymetrix Mapping 50 K *Xba*I and *Hind*III assays had similar resolving powers, so we combined the Xba and Hind data for these analyses.

dChip normalization, scaling and feature extraction provided the highest resolving power for the deletions, with negligible difference between the reference sets of 50 and 214 (Figure 3A). This result indicates that for any given p-value cutoff, on average one would expect to be able to detect the smallest one-copy deletions with dChip feature extraction and our reference set of 50 or 214. Most other methods showed only slightly decreased resolving power. Reference set selection had little effect on resolving power in most cases, although CNAT with the Affymetrix default reference set of 106 ranked the lowest (Figure 3A).

Reference set selection had a greater effect on the resolving power for duplications, and the reference sets chosen from our own data set resulted in higher resolving power than the Affymetrix default set of 106 individuals (Figure 3B). We note that our estimation of the resolving power was probably less accurate for duplications than deletions, since we had a smaller number of confirmed aberrations available for our analysis. Nevertheless, the resolving power was clearly better for deletions than for duplications, such that deletions of a given size could be detected with higher confidence than duplications of the same size.

## Conclusion

We found that CNAG, dChip, CNAT and GLAD were suitable for high-throughput processing of Affymetrix 100 K SNP array data for copy number analysis. Various reference sets selected from data produced by our research team resulted in superior feature extraction, higher signal-to-noise ratios, and higher rates of detection of confirmed CNVs than the external default reference set provided by Affymetrix. This difference may be due to experimental variation between different laboratories, to differences in the frequencies of SNP genotypes and copy number polymorphisms (CNPs) in ethnically diverse populations, or to other unidentified factors. Therefore, we recommend using a reference set, processed in the same laboratory and ideally from samples with a similar ethnic composition to the sample set.

We found considerable variation in the numbers of putative CNVs detected by various software/reference set combinations, and more CNVs were called by dChip than by any other software tested. Rates of false positive deletion calls identified by SNP heterozygosity were substantial with all of the approaches tested, and the false positive call rates did not correlate with the total number of candidate CNVs identified by a given approach. The highest rate of false positive candidate deletion calls was produced by CNAG using a reference set of 2 (within trios), but this is likely, at least in part, due to the very small size of the reference set combined with noisy data. In such a small reference set, the average copy number may be quite different from 2.0 in certain regions of the genome. For example, similar results are expected using pair-wise test versus reference comparisons for the very different cases of a paternally inherited deletion (copy numbers 1, 1, and 2 in the child, father, and mother, respectively) and a duplication in the mother that was not inherited (copy numbers 2, 2, and 3, respectively) (Additional file 16). In such cases, we accepted all possible CNVs as candidates for optimal sensitivity (Methods, Additional file 16), but we expected that a subset of these CNVs would be false positives.

Within a large reference set, the average copy number of all loci is more likely to be close to 2.0, which improves the confidence of CNV detection in a given sample. However, frequent polymorphisms in a large reference set may skew the results. For example, a deletion affecting a single genomic region occurring in 10% of the population could decrease the mean of copy number in that region to 1.9 in a large random reference set, while a deletion with 50% frequency may push the base line to 1.5, resulting in a false positive duplication call in a test sample that lacks the aberration or a false negative deletion call in a test sample that has the deletion. Data from a chromosomal region rich in polymorphic sites might seem noisy and might not yield distinguishable CNVs even though they are frequent. Further understanding of CNVs and their frequencies in the general population will help resolve this issue and increase specificity of CNV detection in these regions.

The performance of software packages in the detection of single copy deletions was better than that of single copy duplications. This may be due to the fact that deletions produce a 2-fold change in copy number (from 2 to 1), while duplications produce only a 1.5-fold change (from 2 to 3).

The rate of detection of confirmed CNVs (Table 4) was independent of the total numbers of CNVs reported (Table 3) by a particular software/reference set combination. As expected, larger CNVs were detected more consistently than smaller ones, and the denser the SNP coverage within a given chromosomal segment, the smaller the CNVs (in bp) that could be detected with high confidence. We found that CNAG-GLAD using pair wise comparisons within trios (reference set of 2) detected the largest proportion (87%) of the 38 validated aberrations, closely followed by CNAT-GLAD with the reference set of 214 (84%) (Table 4). Unfortunately, these two approaches also had the highest false positive deletion rates, of 66% and 51%, respectively (Table 3). No single method detected all of the confirmed CNVs, and each method missed a different subset of variants. Thus, none of the software/reference set combinations we tested appears to be sufficient to detect all true CNVs, and several approaches may need to be used together for efficient and reliable copy number analysis of GeneChip SNP data. For example, to maximize the detection rate of true positive CNVs we recommend using the combination of CNAG-GLAD with pair-wise comparisons of test and reference samples, and the use of dChip and CNAT-GLAD with large reference sets (>50). This combination of approaches successfully detected all of the validated CNVs in our study. To reduce false positive rates, we recommend SNP genotype analysis of putative deletions (see Methods) and setting thresholds for statistical significance and copy number values of putative CNVs.

We used the default parameter settings for copy number analysis in each of the software packages evaluated in this study, as would most users. We did not attempt a thorough parameter optimization due to the large size of the data set and the number of other variables under which the software packages were tested. It should be noted though that changing parameters for some of these software packages may result in different numbers of putative CNVs detected, and the optimal parameters for detection of specific CNVs may also depend on the noise level of each chip and the location and size of the CNVs. Among the packages that we evaluated, there are no applicable parameter settings to CNAT [14,18], and we used CNAT only for normalization and feature extraction, not for CNV detection. dChip automatically determines its optimal HMM parameters for each chip from the raw data [23], thus these parameters are not user accessible. GLAD has a few adjustable parameters for AWS, such as the lambda value for the number of breakpoints and a clustering parameter lambda [26]. We have examined the sensitivity of CNV detection to AWS parameter change from the default on a small subset of samples, and found that the results did not change, even for the detection of our smallest validated aberrations. CNAG's default HMM parameters have been optimized by the software developers to detect full copy number changes (e.g. to 1 or 3) in a mostly diploid sample [22,24]. These parameters are user adjustable, and adjustment is specifically recommended for non-diploid chromosomal regions or detection of mosaic CNVs (with an average copy number change of less than 1.0) (CNAG users' manual [24]). In some cases one may wish to change these parameters to detect as many true CNVs as possible, even though this would also likely produce much higher false positive call rates. In other circumstances, it may be more important to minimize the false positive call rate, even if this means that some real CNVs will be missed. Our study used exclusively samples which are predominantly diploid, and so the default parameters appear the most appropriate.

In addition to the results presented from the empirical data set of 662 arrays, we have also tested the software on a smaller synthetic data set with a higher number of simulated CNVs. Although the numbers of candidate CNVs detected were not directly comparable to those found from empirical data due to the differences between the approaches, the following conclusions were consistent between the empirical and simulation data. Software performance in the detection of deletions was generally better than that of duplications. dChip identified the most putative CNVs from both the empirical and synthetic data. However, it did not have the best true positive CNV detection rate and had significant false positive rate in both cases. On the synthetic data the CNAG-GLAD approach yielded the best true positive CNV detection rate.

The availability of both SNP genotypes and genomic copy number information from the same data is a particularly useful feature of Affymetrix GeneChip Mapping arrays. The copy number analysis algorithms evaluated in this paper all have substantial false positive candidate CNV call rates; however, many putative deletions can be confirmed and a large proportion of false positives can be eliminated without performing further experiments using the genotype information. None of the copy number analysis programs tested here take genotype information into account for identifying candidate deletions, and this would be a useful feature for future implementation. Allelic imbalance in heterozygous genotypes could also be used in calling duplications, as it is in the recently described CARAT algorithm [27].

Another recommendation for improvement of the software packages we tested would be to assign statistical significance to each CNV call and then use this information to rank the candidate CNVs. None of the software packages we tested accurately describes the relative quality of their CNV predictions. An independent statistical test, such as the t-test we employed, is necessary to provide confidence in the CNVs identified by various methods. Furthermore, it would also be useful to rank candidate CNVs by the deviation of copy number from 2.0. A researcher could then decide approximately how many false positive calls to tolerate to achieve the desired rate of true CNV detection by setting the corresponding p-value and copy number thresholds appropriately.

Using a combination of approaches described above to optimize true positive detection rates and minimize false positive rates, we estimated the average numbers of CNVs per genome in our sample set. The average of 39 candidate CNVs in unaffected individuals (20 deletions and 19 duplications) may be an overestimate, because a subset of these may still be false positives. These numbers; however, are in a range similar to those estimated by others in the general population using different technologies and analytical approaches, reviewed by [12]. Many of the CNVs we found in our sample set of 224 unaffected individuals probably represent normal polymorphisms, and future studies will characterize these candidate variants in more detail.

In summary, hybridization data obtained from 100 K SNP WGSA arrays can be used to identify single-copy constitutional CNVs smaller than 200 kb. We found that detecting all real CNVs from such data requires multiple computational approaches. The SNP genotype information that is available for all samples analyzed with these arrays is useful for recognizing many false positive calls and should be used to improve the specificity of CNV detection. Further improvement in the specificity of recognizing true CNVs may be achieved without loss of sensitivity by better use of the information provided by each of the individual 25-mer oligonucleotide probes associated with each SNP on the GeneChip arrays, by taking advantage of the increased resolution of 500 K GeneChip arrays, and by further improving the array design to provide more uniform coverage of the genome.

## Methods

### Affymetrix GeneChip^® ^100 K Mapping Array data

For this analysis, we used a data set generated in a previous study [6] of families with children with mental retardation (MR). The study group was composed of 107 children and both of their unaffected parents, plus 10 unaffected siblings of the affected children from 10 of the families. DNA was isolated from 331 whole blood samples as described [6]. Hybridization to Affymetrix GeneChip^® ^100 K Mapping arrays was performed according to the manufacturer's recommendations (Affymetrix GeneChip^® ^Mapping 100 K Assay Manual; [18]) as previously described [6].

### Reference sets for copy number analysis

The reference sets described below were used for copy number analysis of Affymetrix GeneChip^® ^Mapping 100 K array data:

• '***2***': three pair wise comparisons were performed within each MR trio (child to father as reference, child to mother as reference, and father to mother as reference). Deletions and duplications in family members were called as described in Additional file 16A.

• '***50***': each sample was compared to a reference set of 50 unaffected mothers of children with MR. These 50 mothers selected for this reference set had the smallest numbers of candidate CNVs identified by dChip, compared to the other mothers in our data set (using a reference set of all 214 parents).

• '***214***': each sample was compared to a reference set comprised of all 214 normal parents (107 mothers and 107 fathers) of the children with MR.

• '***106***': a default reference set from 106 individuals provided by Affymetrix for copy number analysis with CNAT [18].

### Synthetic 100 K array data

We generated 30 artificial data sets by randomly shuffling normalized 100 K SNP array data from a normal sample with variability close to the median using the reference set of '50' individuals. Input to the shuffling was normalized copy number data from CNAG, dChip and CNAT, and 10 synthetic samples were produced for each of these software packages. We then introduced 100 simulated CNVs into each synthetic sample with SNP widths ranging from 5 to 23 and copy numbers ranging from 0.3 to 3.0. CNV detection on these normalized data was then performed by dChip and GLAD (this was not possible for the CNAG HMM, since this software does not accept intermediate stage normalized data).

### Copy number analysis with CNAG and CNAG/GLAD

Detection of copy number variants was performed using the Copy Number Analyser for GeneChip^® ^arrays (CNAG) Version 1.1 software [22], using the default parameters. Each sample was compared to a reference set of 2 or 50 individuals. Regions of copy number gains or losses were detected using the Hidden Markov Model (HMM) output of CNAG. Deletions and duplications in individuals were identified using the rules described in Additional file 16AA (for the reference set of 2) and B (for the reference set of 50).

In addition to the CNAG HMM implementation, we also identified copy number changes using the Gain and Loss Analysis of DNA (GLAD) R package [26] with default settings. Sample versus reference SNP copy number log_2_-ratios calculated by CNAG were used as the input for the CNAG/GLAD analysis.

### Copy number analysis with dChip and dChip/GLAD

Genomic gains and losses were assessed against the reference sets of 50 or 214 individuals using the DNA-Chip Analyzer (dChip) Version Release (Nov 17 2005) software [23] with the default parameters. Regions of copy number gain or loss in each comparison were detected using the Hidden Markov Model (HMM) output of dChip.

We also detected copy number changes using the GLAD R package [26] with the default settings instead of the dChip HMM. SNP copy number log_2_-ratios of sample versus reference calculated by dChip served as the input for the dChip/GLAD analysis. Deletions and duplications in individuals were identified following the rules described in Additional file 16B.

### Copy number analysis with CNAT and GLAD

SNP copy numbers were determined using the Affymetrix GeneChip^®^ Chromosome Copy Number Analysis Tool (CNAT) Version 3.0 [14,18] using the default parameters and the reference sets of 50, 214 or 106 individuals. We used the GLAD R package (Hupe et al. 2004) to identify CNV boundaries from SNP copy number log_2_-ratios of sample versus reference sample set calculated by CNAT. Deletions and duplications in individuals were identified using the rules described in Additional data file 16B.

### Genotype analysis of deletions

SNP genotype calls were generated from probe signal intensity data using the GeneChip^® ^DNA Analysis Software Version 3.0 (GDAS) [18], with a confidence score threshold of 0.05 for genotype accuracy. We counted the number of heterozygous SNPs within putative deletions identified by each copy number analysis method described above. If the rate of heterozygous SNPs was more than 10% of the total SNP count within a candidate deletion, the deletion was considered to be a false positive call. If no more than 10% of SNPs were heterozygous, the deletion was accepted. One reason for allowing up to 10% heterozygous SNP call rate (instead of 0%) within deletions was the occasional occurrence of errors in genotype calls. Furthermore, although the presence of a deletion may correctly be identified in a chromosomal segment by a software package, the breakpoints may not be accurately defined, resulting in the inclusion of heterozygous SNPs from the normal region(s) flanking the deletion boundaries. The percentage of heterozygous SNPs was below 10% in all of our validated deletions. All candidate deletions with more than 10% SNP heterozygosity that we attempted to validate turned out to be false positives.

### Validation of putative copy number variants (CNVs)

Validation of most putative CNVs was carried out by fluorescent *in situ *hybridization (FISH) on interphase and metaphase chromosome spreads prepared according to standard cytogenetic protocols, as described [6]. Bacterial artificial chromosome (BAC) or fosmid inserts were used as probes, selected via the University of California at Santa Cruz (UCSC) genome browser [28,29], May 2004 human genome assembly. A subset of CNVs were confirmed using standard karyotyping, and one inherited deletion was validated using quantitative PCR as previously described [30].

### Theoretical resolving power for detecting hemizygous deletions and duplications

We defined the resolving power as the average size of the smallest one-copy deletion or duplication that could be detected at a given confidence level. The confidence level of finding hemizygous deletions (1 copy) or duplications (3 copies) containing **n **number of SNPs from feature extracted copy number data by various methods was estimated using the Welch t-test as follows. SNP copy number log_2_-ratios of sample versus reference were calculated from probe intensity values using CNAT, CNAG or dChip. The means and standard deviations of these ratios were calculated within each validated CNV, and in the rest of the same chromosome outside the CNV that we used as the control region. Assuming that the mean and standard deviation of any 2 or more SNPs chosen from within the CNV or control region would be equal (in keeping with a Gaussian distribution), we compared the average log_2_-ratios between **n **SNPs from the CNV with (**c-n) **SNPs from the control region, where **'c' **represents the total number of SNPs for that chromosome on the array. Using the Welch t-test, a p-value was calculated. The resolving power for deletions and duplications were calculated from combined Xba and Hind data using the average values of confirmed deletions and duplications, respectively, and then by extrapolating to calculate p-values required to detect a wide range of potential CNV sizes.

## Abbreviations

Array-CGH, array comparative genomic hybridization; AWS, adaptive weights smoothing; CNP, copy number polymorphism; CNV, copy number variant; FISH, fluorescent *in situ *hybridization; HMM, Hidden Markov Model; MR, mental retardation; SD, standard deviation; SNP, single nucleotide polymorphism; WGSA, whole genome sampling analysis

## Competing interests

The author(s) declares that there are no competing interests.

## Authors' contributions

AB was involved in the study design, generation of 100 K SNP array data, 100 K SNP array data analysis, and wrote and edited the manuscript. ADD participated in the study design, 100 K SNP array data analysis and manuscript editing. HIL, TN, SF and HQ performed 100 K SNP array data analysis and manuscript editing. SYC participated in generation of the 100 K SNP array data and manuscript editing. JA, AA, MC, MB-J, AG and GK generated 100 K SNP array data. PB, NF, and SL were involved in collecting patient samples. PE carried out validation of putative CNVs. JMF was involved in patient sample collection and manuscript editing. MAM participated in the study design, supervised the study and edited the manuscript. All authors read and approved the final manuscript.

## Additional files

Raw array data are publicly available within the NCBI Gene Expression Omnibus (GEO) database [31] under accession number GSE 7226. The raw data can also be downloaded from  using the login: mr and password: omn1w0rld.

## Supplementary Material

Additional file 1 List of samples and oligonucleotide arrays. List of 331 samples and 662 arrays (Xba 50 K and Hind 50 K) with corresponding array quality measures. Click here for file

Additional file 2 Candidate CNVs by CNAG Ref2. List of candidate copy number variants identified by CNAG using reference set '2'. Click here for file

Additional file 3 Candidate CNVs by CNAG Ref50. List of candidate copy number variants identified by CNAG using reference set '50'. Click here for file

Additional file 4 Candidate CNVs by CNAG-GLAD Ref2. List of candidate copy number variants identified by CNAG and GLAD using reference set '2'. Click here for file

Additional file 5 Candidate CNVs by CNAG-GLAD Ref50. List of candidate copy number variants identified by CNAG and GLAD using reference set '50'.> Click here for file

Additional file 6 Candidate CNVs by dChip Ref50. List of candidate copy number variants identified by dChip using reference set '50'. Click here for file

Additional file 7 Candidate CNVs by dChip Ref50 (continued). List of candidate copy number variants identified by dChip using reference set '50' (continued). Click here for file

Additional file 8 Candidate CNVs by dChip Ref214. List of candidate copy number variants identified by dChip using reference set '214'. Click here for file

Additional file 9 Candidate CNVs by dChip-GLAD Ref50. List of candidate copy number variants identified by dChip and GLAD using reference set '50'. Click here for file

Additional file 10 Candidate CNVs by dChip-GLAD Ref214. List of candidate copy number variants identified by dChip and GLAD using reference set '214'. Click here for file

Additional file 11 Candidate CNVs by CNAT-GLAD Ref50. List of candidate copy number variants identified by CNAT and GLAD using reference set '50'. Click here for file

Additional file 12 Candidate CNVs by CNAT-GLAD Ref106. List of candidate copy number variants identified by CNAT and GLAD using reference set '106'. Click here for file

Additional file 13 Candidate CNVs by CNAT-GLAD Ref214. List of candidate copy number variants identified by CNAT and GLAD using reference set '214'. Click here for file

Additional file 14 Candidate CNVs predicted by multiple methods. Putative CNV regions identified by at least two software/reference set combinations from the same sample. Click here for file

Additional file 15 Detection of confirmed CNVs. List of 38 CNVs that were confirmed by independent experimental approaches, as well as their detection by various software/reference set combinations. Click here for file

Additional file 16 Rules for candidate CNV detection. The rules used for the detection of putative copy number variants with various reference sets. Click here for file
